# An exploration of whether the extent and orientation of the discrepancy in perceived and actual mathematical efficacy affects mathematical anxiety

**DOI:** 10.3389/frcha.2022.1041662

**Published:** 2023-01-10

**Authors:** Wang Xi li, Li Xue Liu, Mu Xia

**Affiliations:** ^1^ College of Education Science, Guangxi Minzu University, Nanning, China; ^2^ Fujian Polytechnic of Information Technology, Digital Industry College, Fuzhou, China; ^3^ School of Physical Education, Guangxi Normal University, Nanning, China

**Keywords:** self-Assessment, mathematical efficacy, bias, mathematics anxiety, high-school students

## Abstract

A questionnaire survey was used to study the effect of the discrepancy between perceived and actual mathematical efficacy (discrepancy in mathematical efficacy) on mathematics anxiety, and the moderating effect of bias in mathematical efficacy estimation. A total of 582 grade 1 students in seniors high school in Nanning with an average age of 16 and 489 grade 2 students in seniors high school with an average age of 17 were selected. The results confirmed that (1) mathematics anxiety was significantly negatively correlated with mathematics efficacy, and significantly positively correlated with greater discrepancy between perceived and actual mathematics efficacy. (2) Variation in mathematics efficacy was much greater, given underestimation rather than overestimation of efficacy. Further, mathematics anxiety was significantly greater, given underestimation vs. overestimation of efficacy. (3) Discrepancy in perceived vs. actual mathematical efficacy positively predicted mathematics anxiety, and was moderated by bias in the estimation of mathematical ability. In the first-grade 1 students, mathematics anxiety was predicted by underestimation but not overestimation of efficacy.

## 1. Introduction

As one of the core courses of China's basic education system, mathematics tests students' logical thinking ability and is crucial to the intellectual development and academic development of high school students. Learning anxiety not only affects students' overall academic performance, but also their future career choices and long-term development prospects (1, 2). Research on mathematics anxiety outside of China began in the 1950 s (3), however, the definitions of anxiety were not uniform. Mathematics may produce a negative emotional response in students, and this anxiety response generally leads to students' low mathematics achievement. Namkung et al. (4) defined math anxiety as the feeling of panic or anxiety that an individual experiences when encountering and using related math problems in life and school situations. Such feelings interfere with students' mathematical operations, lead to poor performance in addressing mathematical problems, affect their willingness to pursue higher mathematics development, and then affect their overall academic and career development. Research on mathematics anxiety in China began later than that in other countries; Chinese research began in the 21st century, with researchers such as Yinghe (5) suggesting that mathematics anxiety refers to anxiety states such as panic, anxiety, and fear generated in the process of acquiring mathematics, taking mathematics examinations, using numbers, and using mathematics concepts.

Several surveys and analyses have revealed that Chinese middle-school students generally experience math anxiety. For example, Juanjuan (6) reported different levels of math anxiety in the three grades of a high school, among which high school students exhibited a moderate-to-high level. Fei (7) found that 8.8% of high school students exhibit severe anxiety and are in urgent need of help. Thus, mathematics anxiety among middle-school students is a common negative emotion. Appropriate anxiety and stress can help improve students' learning efficiency, but excessive anxiety not only leads to a decline in academic performance, but also to a series of psychological problems, such as neurotic anxiety disorder. Therefore, it is important to explore the internal mechanisms that influence middle-school students' mathematics anxiety, to help such students reduce their mathematics anxiety from the cause and improve their level of mathematics ability.

Mathematical self-efficacy is a specific reflection of self-efficacy theory in the category of mathematics, which belongs to a category of academic self-efficacy. Betz and Hackett (8) specifically defined mathematical efficacy as follows: “Mathematical efficacy is the individual”s confidence in his ability to successfully complete and solve certain (mathematical) tasks, problems or setbacks, making problem-specific and situational judgments.” A recent study of the career interests of middle-school students found that self-efficacy affects their future career choices (9); another study found that self-efficacy is crucial to academic performance (10), and is a major predictor of academic achievement (11, 12). Shuyuan and Zhijian (13) confirmed that college students with high academic self-efficacy may experience relatively more positive emotions associated with academic efforts. This observation was supported in high-school students by Xiancai (14). Cultivating students' positive attitudes, emotions, and beliefs in mathematics is crucial for mathematics learning (15, 16). Thus, self-efficacy has an important impact on students’ academic mood and future development.

Although there are few studies of mathematics efficacy and mathematics anxiety, those available have revealed a strong correlation between mathematics efficacy and mathematics anxiety (17, 18). When summarizing the relationship between mathematics self-efficacy and mathematics anxiety, Chinese researchers have reported that mathematics self-efficacy can indirectly affect students' academic performance through anxiety and metacognition (19). Further, international researchers have found that mathematics self-efficacy is one factor that affects mathematics anxiety. A positive self-concept regarding higher mathematics ability can significantly reduce the individual's mathematics anxiety (20), and the correlation between mathematics anxiety and mathematics self-efficacy is stronger than that between mathematics anxiety and value beliefs—intrinsic value and achievement value (21). Bartley and Ingram (22) found that mathematics self-efficacy directly and negatively predicted mathematics anxiety in a predictive model of mathematics anxiety among high school students. From the above studies, it appears that the greater one's efficacy, the greater the inhibitory effect on math anxiety. However, the question then arises as to whether such performance is truly advantageous.

In this respect, Bandura et al. (23) suggested that a greater sense of efficacy is universally better, but there needs to be some consistency between the sense of efficacy and the actual performance of the individual. Overestimating one's own ability will make the individual lose the opportunity for development, while overestimating one's own ability likely lead to failure; thus, it is best to slightly overestimate one's own abilities. Taylor and Brown (24) also suggested that an optimistic estimate of one's own abilities is a normal feature of the human mind that helps people adapt, create, and respond to constructive criticism in a positive way. However, overconfidence, or a mismatch between efficacy judgments and performance, is less beneficial because it can lead to poor preparation and a lack of self-awareness regarding weaknesses that need to be addressed. Pajares and Miller (25) concluded that most students inaccurately judge their own efficacy, and hence proposed the concept of efficacy deviation. This refers to the absolute difference between judgments of self-efficacy and actual ability; the greater the value, the greater the inconsistency between the judgment and the actual ability. According to research on the relationship between self-efficacy judgments and mathematics performance by domestic and international researchers, the more accurate the efficacy judgment, the better the mathematics performance (26–28). Zimmerman, Bonner and Kovach (29) found that when the deviation from true efficacy is too large, students are blindly confident, persist with their default learning style and are reluctant to make changes, resulting in a decline in performance. Schumann and Sibthorp (30) also found that the greater the deviation of teachers’ perceived teaching self-efficacy from their actual ability, the lower the teaching quality. García and Fidalgo (31) found that with respect to the writing efficacy of students with learning disabilities, the greater the discrepancy between students' perceived and actual writing efficacy, the less prepared they were and the lower their writing quality. Talsma, Schüz and Norris (32) found that among college students, an excessive discrepancy in academic self-efficacy can negatively impact academic self-regulation and performance.

In summary, the greater the individual's self-efficacy, the better their behavioral performance. However, when the discrepancy between perceived and actual self-efficacy is too large, behavioral performance becomes worse. Therefore, the difference between perceived mathematical self-efficacy and actual mathematical ability, namely the degree of discrepancy in mathematical efficacy, should also influence mathematics anxiety. However, the discrepancy in efficacy can be further divided into overestimation and underestimation. Overestimation denotes an optimistic view of one's actual ability, namely one's self-efficacy judgment is greater than the actual ability. In contrast, underestimation denotes a negative view of one's actual ability, such that self-efficacy judgments are lower than one's actual ability. When individuals overestimate their abilities, the greater the discrepancy between perceived and actual self-efficacy; in this situation, there is likely a negative correlation between the degree of discrepancy in mathematics efficacy and mathematics anxiety. In contrast, when individuals underestimate their own abilities, the greater the discrepancy between perceived and actual self-efficacy, there is likely a positive correlation between the degree of discrepancy in mathematics efficacy and mathematics anxiety.

**Figure 1:**
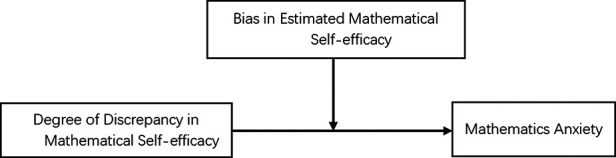
Hypothetical model of the moderating effect of biased judgment of mathematic self-efficacy on the relationship between the degree of discrepancy in self-assessed vs. actual math efficacy and mathematics anxiety.

However, previous studies have not explored whether the extent of discrepancy has different effects on anxiety given overestimation and underestimation. According to Pajares' measure of discrepancy in mathematical efficacy (25), this study refers to such overestimation and underestimation as efficacy estimation bias. Accordingly, we explored the influence of the variations in mathematics efficacy on mathematics anxiety and the moderating effect of mathematical efficacy estimation bias. Assumption model is shown in Figure 1.

## 2. Materials and methods

### 2.1. Participants

On a voluntary basis, the data were collected *via* a questionnaire survey conducted in a senior middle-school in Nanning City. From 582 grade 1 in senior high school, with an average age of 16, 576 valid questionnaires were collected, and from 489 grade 2 in senior high school, with an average age of 17, a total of 473 valid questionnaires were collected.

### 2.2. Research tools

#### 2.2.1. Mathematical anxiety scale

The Mathematical Anxiety Scale compiled by Bai et al. (33) and translated by Wei (34) was adopted. The questionnaire assesses a single dimension *via* eight items, each of which is rated using a five-point Likert scale (1 = completely disagree, 5 = completely agree). The higher the score, the more anxious the respondent with respect to learning mathematics. The Cronbach's alpha of the scale was 0.926.

#### 2.2.2. Math test paper

Topics were selected from the mathematics test paper of the large-scale joint entrance examination at the end of the middle-school semester, to ensure that the test was realistic for the students and would correctly reflect their psychological state. Due to the limited test time and the need to combine tasks with different levels of difficulty in the efficacy test, six questions were selected as the mathematics test paper, with three levels difficulty: easy, medium, and difficult. The exam paper for grade 1 in seniors school. students consisted of five multiple-choice questions and one fill-in-the-blank question, whereas the second-year exam paper consisted of four multiple-choice questions and two fill-in-the-blank questions. Sixteen points were awarded for a correct answer to a question and 1 point for an incorrect answer. This is to correspond with the self-efficacy score to facilitate the calculation of the difference between an individual's math self-efficacy and actual math ability.

The difficulty Cronbach's alphas of the six math questions for the grade 1 in seniors school group were 0.95, 0.85, 0.6, 0.56, 0.27, and 0.2. The difficulty coefficients of the six math questions for the grade 2 in seniors school group were 0.85, 0.91, 0.65, 0.68, 0.38, and 0.33. Therefore, in both grades, the 1st and 2nd questions were easy, the 3rd and 4th questions were moderately difficult, and the 5th and 6th questions were difficult.

#### 2.2.3. Mathematical efficacy

The Mathematics Efficacy Scale proposed by Pajares and Miller (25) and revised by Siyu (26) was used to assess mathematical self-efficacy. The students first saw the mathematical test questions and then were asked to judge “whether you can answer this question with ease.” The mathematical efficacy questionnaire included two dimensions, outcome efficacy and degree of self-efficacy. The questionnaire uses a four-point Likert scale scoring method (1 = completely disagree, 4 = agree very much). Analysis showed that the internal consistency coefficients of the two questions in the six-question math exercises were 0.874 and 0.868.

Outcome efficacy measured how easily the respondents could answer the questions in practice, whereas the degree of self-efficacy measured the respondents' subjective perception of their ability to answer each question. Individuals are confident in their mathematical ability only if they believe they can answer mathematical question easily. Therefore, we referred to the method of calculating achievement motivation in Atkinson's expected value theory (35). We determined that calculation of an overall efficacy score should be the product of result efficacy score and the degree of self-efficacy score. Only when both scores are high is the individual's sense of efficacy high. If an individual believes they can answer the question correctly but the process of generating the answer is not easy, or if the individual believes they cannot answer the question correctly and process of generating the answer was not easy, the overall efficacy of the subjects is relatively low. For example, if the maximum score of each rating 10, if an individual's result efficacy score is eight and their degree of self-efficacy score is 0.5, an additive overall efficacy would be 8.5 points, vs. a multiplicative overall efficacy score of four points. That is, multiplication better reveals the effect of result efficacy and the degree of self-efficacy on overall efficacy. Since outcome efficacy and degree of self-efficacy scores both ranged from 1 to 4, the total mathematical efficacy score ranged from 1 to 16.

### 2.3. Measures of discrepancy in mathematical efficacy and estimation bias

The degree of discrepancy in efficacy refers to the absolute value of the difference between an individual's efficacy score and their actual ability level. This score was taken as absolute value of the total individual efficacy score minus the total score of objective indicators corresponding to the actual ability of the individual. For example, if an individual's assessment of self-efficacy for a simple math problem was two points and the result efficacy was 4 points, and the individual also obtained the correct answer and thus received 16 points, then the discrepancy in mathematic efficacy would be |(2 × 4) − 16| = 8. If the result is negative before taking the absolute value, the mathematical efficacy is underestimated. If the result is positive before taking the absolute value, the mathematical efficacy is overestimated. Scored ranged from 0 to 15; the mathematical efficacy estimation bias was defined as the value one for underestimation, and two for overestimation.

### 2.4. Research process

In the questionnaire, the Mathematical Anxiety Scale and Mathematical Efficacy Scale were on one side of the paper and the math test questions on the other side. Before the formal distribution of the questionnaire, several students in the first and second grades of the senior high school were invited to help test the time required to complete the questionnaire. Under the condition of strict compliance with the instructions, it required approximately 13 min for the students to complete both sides of the questionnaire. The complete test time varied from 20 min to 60 min.

The questionnaire was administered during students' self-study period, using consistent instructions. The students were asked to complete the questionnaire, consisting of the Mathematical Anxiety Scale and the Mathematic Efficacy Scale in turn, according to their actual situation; the students were informed that there were no correct or incorrect answers. Questionnaires were completed independently. The students were told that when completing the efficacy question, to only consider whether the first question could be answered correctly. If the student believed that there was a high probability that they could not provide the correct answer, they were to select the “completely impossible” option for the second question. After explaining the instructions, the main test administrator distributed the questionnaires. After the students had completed the questionnaires, the administrator collected the questionnaires.

Since the Mathematical Efficacy Scale is a specific task judgment, to prevent the students from calculating the answers to the mathematical questions while completing the efficacy measurement, the time allowed for students to complete the first questionnaire was strictly controlled. Further, it was emphasized that mathematical problems should not be solved while completing the efficacy questions. All questionnaires were to be collected within 13 min, to the extent possible.

After a period, all of the participants were asked to complete a mathematics test paper consisting of multiple-choice questions and fill-in-the-blank questions. The participants were informed that the score on this paper were unrelated to their scores on the prior test. The participants answered the questions independently. To reduce any possible effects of time allotted, 20 min was set as the upper limit to complete the paper.

## 3. Results

In this study, SPSS 22.0 was used to generate descriptive statistics, correlations, differences, and regression analyses for each variable. The PROCESS 4.0 macro for SPSS was used to test the moderating effect of discrepancy in mathematical efficacy, estimation bias, and mathematical anxiety. Due to the different mathematics test papers used with the grade 1 in seniors school and grade 2 in seniors school groups, the mathematics efficacy scores could not be aggregated for analysis. Therefore, data analysis was carried out separately for the grade 1 in seniors school and 2 in seniors school students.

### 3.1. Descriptive statistics

Tables 1, 2 show that among the first and second grade students, there was a significant negative correlation between mathematics anxiety and mathematics efficacy. In the first grade of senior high school, the correlation between mathematical self-efficacy and mathematical anxiety reached −0.468; In the second year of senior high school, the correlation between mathematical self-efficacy and mathematical anxiety reached −0.471; a significant positive correlation between the degree of discrepancy in mathematics efficacy, In the first grade of senior high school, the correlation between mathematical anxiety and mathematical efficacy deviation reached 0.12; In the second year of senior high school, the correlation between mathematical anxiety and mathematical efficacy deviation reached 0.323; and a significant negative correlation between mathematical efficacy and the degree of discrepancy in mathematics efficacy, In the first grade of senior high school, the correlation between the sense of mathematical efficacy and the deviation degree of mathematical efficacy reached −0.176; In the second grade of senior high school, the correlation between mathematical efficacy and mathematical deviation reached −0.649.

**Table 1:** Mean, standard deviation, and correlation for each variable in the grade 1 in seniors school. cohort.

	*M*	SD	*N*	1	2	3
1. Math anxiety	21.88	7.94	576	1		
2. Efficacy	50.90	23.24	576	−0.468*	1	
3. Degree of Discrepancy in Efficacy	35.06	15.16	576	0.120*	−0.176*	1

* *p* < 0.01.

**Table 2:** Mean, standard deviation, and correlation of each variable in the grade 2 in seniors school. cohort.

	*M*	SD	*N*	1	2	3
1. Math anxiety	20.48	7.42	473	1		
2. Efficacy	62.98	24.67	473	−0.471*	1	
3. Degree of Discrepancy in Efficacy	33.52	18.85	473	0.323*	−0.649*	1

* *p* < 0.01.

### 3.2. Difference analysis

To determine whether the observed and expected frequencies of the two categories of high school students' mathematical efficacy estimation bias were consistent, a chi-square test was performed separately for the high school students in the first and second grades. The results are shown in Tables 3, 4. The mathematics performance estimates of grade 1 in seniors school. and grade 2 in seniors school. students tended toward underestimation rather than overestimation.

**Table 3:** Chi-square test for bias in estimated mathematical efficiency in grade 1 in seniors school. cohort.

	Observations, *N*	Expected value, *N*	Residual	Chi-square	*df*	*t*
Underestimated	399	281.5	117.5	98.091^a^	1	<0.001
Overestimated	164	281.5	−117.5			
Total	563					

^a^ No variables had a frequency <5.

**Table 4:** Chi-square test of bias in estimated mathematical efficacy in grade 2 in seniors school. cohort.

	Observations, *N*	Expected value, *N*	Residual	Chi-square	*df*	*t*
Underestimated	267	214.5	52.5	25.699^a^	1	0.000
Overestimated	162	214.5	−52.5			
Total	429					

^a^ No variables had a frequency <5.

**Figure 2:**
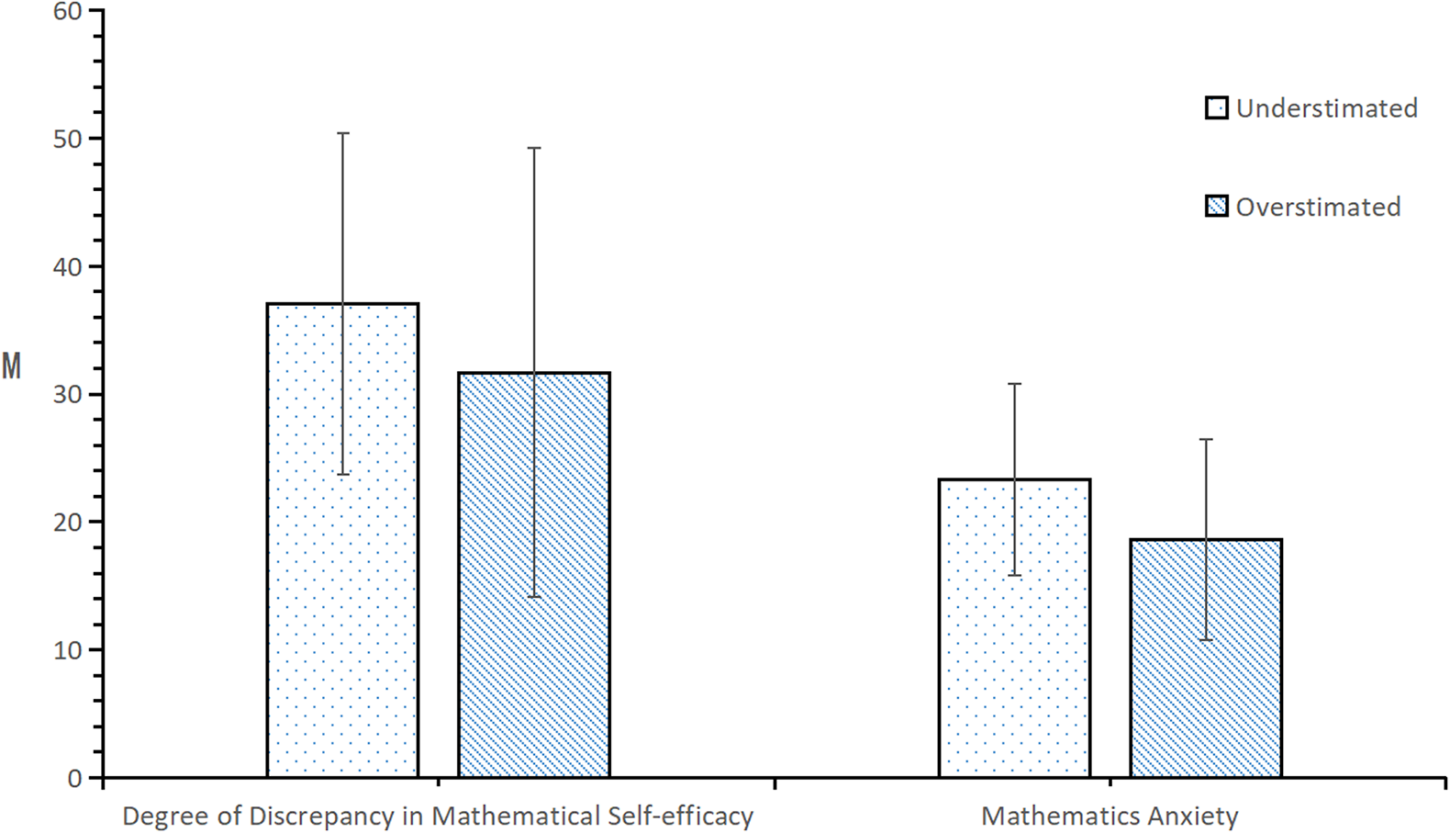
Test of differences in the degree of discrepancy in mathematics efficacy and mathematics anxiety according to the estimation bias of high-school students.

From the results in Figure 2, it can be seen that in the grade 1 in seniors school group, the degree of discrepancy in mathematical efficacy was significantly different between the mathematical efficacy estimation bias groups (*t* = 3.941, *p* < 0.001). The degree of underestimation was much greater than the degree of overestimation. There was a significant difference in mathematics anxiety level between the estimation bias groups (*t* = 6.625, *p* < 0.001), such that mathematics anxiety was significantly greater among those who underestimated rather than overestimated their mathematics efficacy.

**Figure 3:**
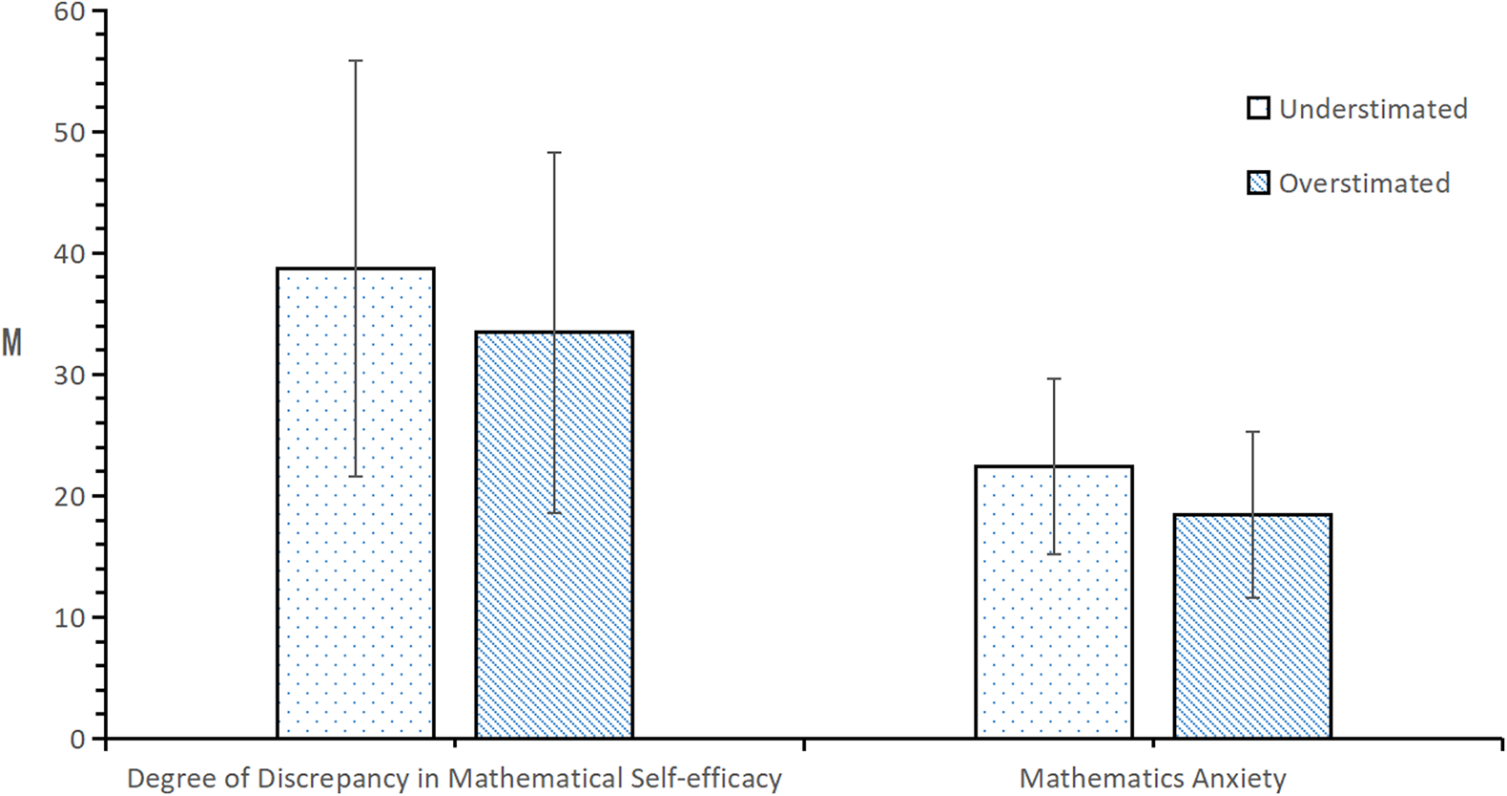
Test of differences in the degree of discrepancy in mathematics efficacy and mathematics anxiety according to the estimation bias of high-school sophomores.

From the results in Figure 3, it can be seen that in the senior of the two groups, the degree of discrepancy in mathematical efficacy was significantly different between the mathematical efficacy estimation bias groups (*t* = 3.242, *p* < 0.01). The degree of underestimation was much greater than that in the younger group. Mathematics anxiety significantly varied by estimation bias group (*t* = 5.668, *p* < 0.001), such that anxiety was significantly greater among those who underestimated rather than overestimated their mathematics efficacy.

### 3.3. Moderating effect test

**Figure 4:**
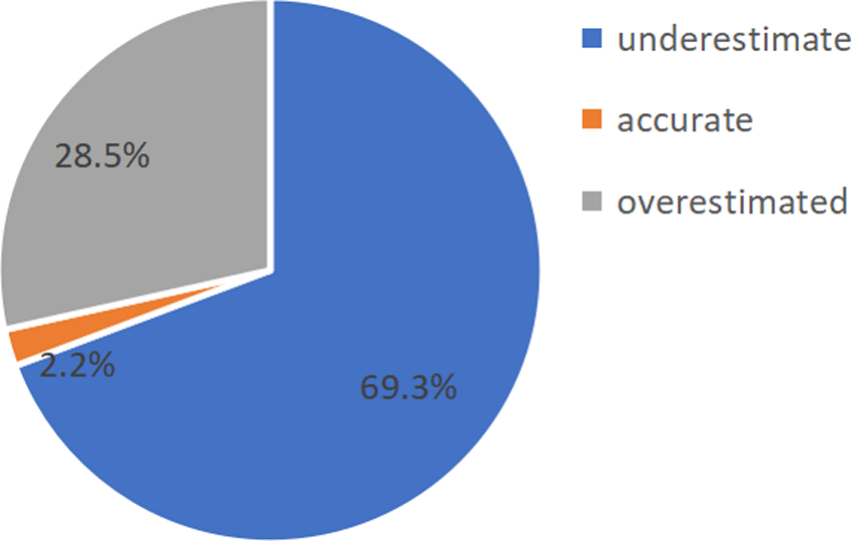
Proportion of grade 1 in seniors school. high-school students with different efficacy estimation bias.

**Figure 5:**
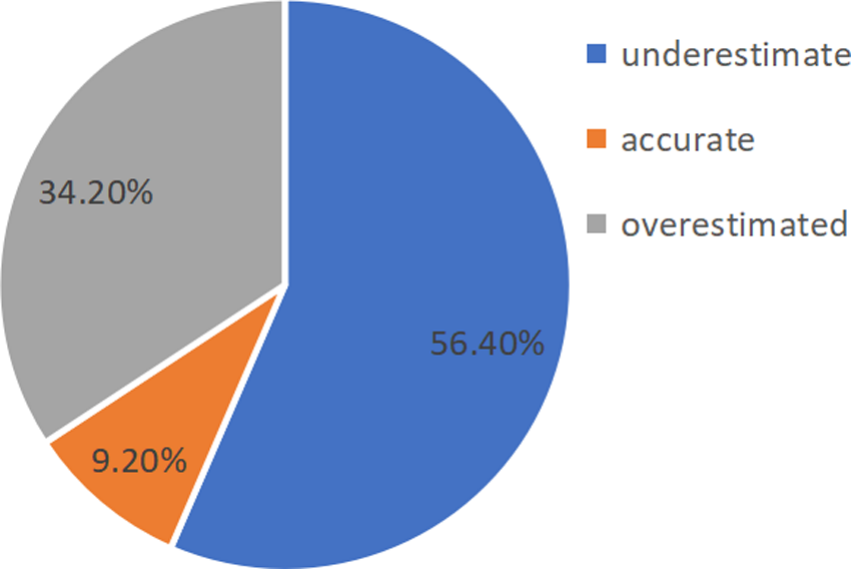
Proportion of grade 2 in seniors school. high-school students with different efficacy estimation bias.

According to the results in Figures 4, 5, there were few cases of accurate judgments of efficacy among the grade 1 in seniors school and 2 students. As the focus of this study was to explore the moderating role of mathematical efficacy overestimation and underestimation and the effect of discrepancy in mathematical efficacy on mathematics anxiety, the cases that of accurate judgments were removed before the moderating effect analysis was carried out. Thirteen cases were deleted from the grade 1 in seniors school. dataset, leaving 563 cases, and 44 cases were deleted from the grade 2 in seniors school. dataset, leaving 429 cases.

A moderating effect analysis was performed on the estimation bias of mathematics efficacy among the grade 1 in seniors school students. The results are shown in Table 5. For these students, the overall model was significant, *F* = 19.813, *p* < 0.001, Δ*R*^2^ = 0.096. The main effect of the degree of discrepancy in mathematical efficacy was significant (*t* = 2.761, *p* < 0.001), as was the main effect of bias of mathematical efficacy estimation (*t* = −6.600, *p* < 0.001), and the interaction between the two variables (*t* = −3.340, *p* < 0.01). In general, an interaction between the degree of discrepancy in mathematical efficacy and mathematical anxiety, and the mathematical efficacy estimation bias had a significant moderating effect.

**Table 5:** Moderation analysis of the effect of bias in estimated mathematics efficacy of first graders on the degree of discrepancy in mathematics efficacy.

Predictor	*B*	SE	*t*	95% CI	
Efficacy discrepancy	0.247	0.066	2.761***	0.118	0.377
Efficacy estimation bias	−4.689	0.710	−6.600***	−6.084	−3.293
Efficacy discrepancy × Efficacy estimation bias	−0.147	0.044	−3.340**	−0.233	−0.060
*R* ^2^			0.310		
Δ*R*^2^			0.096		
*F*			19.813 ***		

Further simple effects analysis was carried out on the mathematical efficacy estimation bias. It can be seen from Table 6 that in the first grade, mathematical efficacy estimation bias significantly moderated the influence of discrepancy in mathematical efficacy on mathematics anxiety. In the case of underestimation of mathematical efficacy, there may be a positive interaction between the degree of discrepancy in mathematical efficacy and mathematical anxiety (*t* = 3.546, *p* < 0.001), whereas in the case of overestimation of mathematical efficacy, the degree of discrepancy is no significant correlation with mathematics anxiety (*t* = −1.362, *p* = 0.174).

**Table 6:** Simple effects analysis of the role of bias in the adjusted model of mathematical efficacy estimation bias of high-school students.

Mathematical efficacy estimation bias	Effect size	SE	*t*	LLCI	ULCI
Underestimated	0.101	0.028	3.564***	0.045	0.156
Overestimated	−0.046	0.034	−1.362	−0.112	0.020

**Figure 6:**
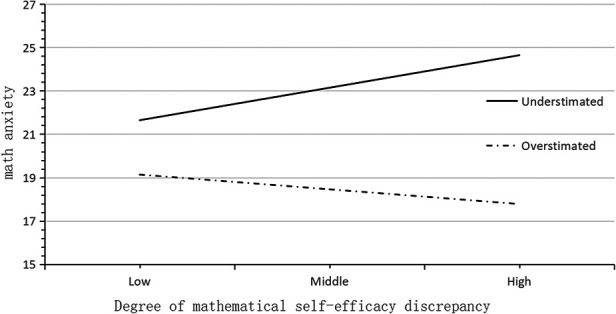
Simple slopes plot of math efficacy estimation bias among grade 1 in seniors school. students.

**Figure 7:**
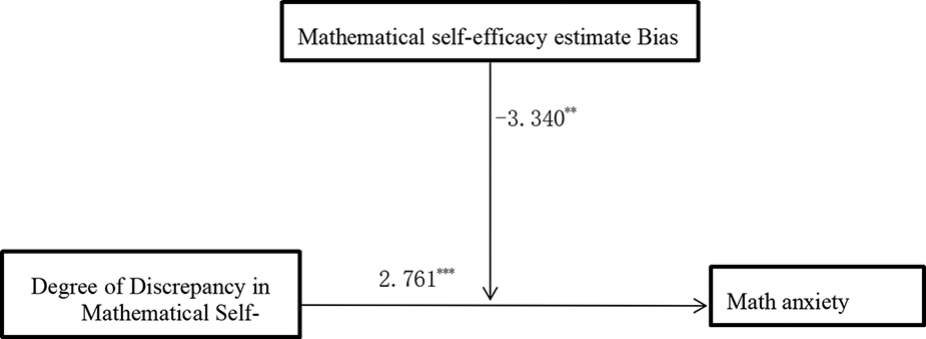
Moderation model of the effect of the degree of discrepancy in mathematics efficacy among high-school students on mathematics anxiety.

A simple slopes diagram depicting the role of bias in mathematics efficacy estimation can be seen in Figure 6. Among the first-year students who underestimated their mathematics efficacy, their mathematics anxiety maybe increased with increasing discrepancy in mathematics efficacy. For students who overestimated their mathematics efficacy, mathematics anxiety maybe decreased with increasing discrepancy in mathematics efficacy. A moderation effect model diagram is shown in Figure 7.

A similar moderating effect analysis was carried out on the estimation bias in mathematics efficacy of the second-year students. The results are shown in Table 7. The overall model was significant, *F* = 19.427, *p* < 0.001, Δ*R*^2^ = 0.121. The main effect of the degree of discrepancy in mathematical efficacy was not significant (*t* = 1.325, *p* = 0.186), the main effect of bias in mathematical efficacy estimates was significant (*t* = −4.919, *p* < 0.001), and the interaction between the two variables was not significant (*t* = 0.337, *p* = 0.736). In general, the degree of discrepancy in mathematical efficacy did not predict mathematical anxiety, and the moderating effect of mathematical efficacy estimation bias was not significant.

**Table 7:** Moderation analysis of the effect of bias in estimated mathematics performance of first graders on the degree of discrepancy in mathematics performance.

Predictor	*B*	SE	*t*	95% CI	
Efficacy discrepancy	0.081	0.061	1.325	−0.039	0.202
Efficacy estimation bias	−3.440	0.669	−4.919***	−4.815	−2.065
Efficacy discrepancy × Efficacy estimation bias	0.015	0.044	0.337	−0.072	0.102
*R* ^2^			0.347		
Δ*R*^2^			0.121		
*F*			19.421		

**Figure 8:**
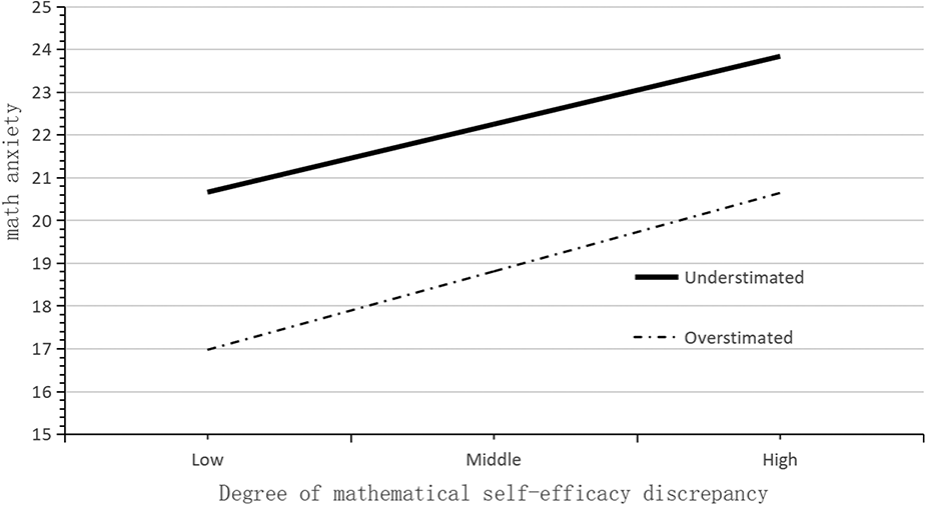
Simple slopes plot of bias in mathematics efficacy estimates of high-school students.

A simple slopes diagram of the role of estimation bias of mathematics efficacy is depicted in Figure 8. It can be seen that among the students in the second grade of senior high school, the influence of the degree of discrepancy in mathematics efficacy on mathematics anxiety was not moderated by the estimation bias of mathematics efficacy. The slopes of the relationship between discrepancy in mathematical performance and mathematics anxiety were almost parallel for cases of overestimation and underestimation.

In summary, the model proposed in this study, in which mathematical efficacy estimation bias played a moderating role in the effect of discrepancy in mathematical efficacy on mathematics anxiety was supported for the first-year students, but not for the second-year students. There is a positive interaction between the degree of discrepancy in mathematical efficacy and mathematical anxiety in first-year high school students. When mathematics efficacy was underestimated, the influence was enhanced; that is, a greater degree of underestimation was associated with more severe mathematics anxiety. However, overestimation did not act as a moderator; that is, when the students overestimated their mathematics efficacy, the degree of discrepancy in mathematics efficacy no longer affected mathematics anxiety. In the group of grade 2 in seniors school. students, regardless of whether mathematical efficacy was overestimated or underestimated, it did not affect the relationship between the degree of discrepancy in mathematical efficacy and mathematics anxiety.

## 4. Discussion

### 4.1. Differences between discrepancy in mathematics efficacy and mathematics anxiety according to estimation bias

More students underestimated than overestimated their mathematics efficacy, regardless of school year. High-school first and second year students are lacking confidence in their actual mathematical ability, likely because high school mathematics is relatively difficult, and it is challenging to obtain high grades or full marks. Brown (36) aimed to evaluate students' strengths, whereas the current study considered at the actual level of mathematical ability. Mathematics is not the best subject for all students. On the contrary, in high school, mathematics is considered a more difficult subject, which tests students' logical thinking and reasoning abilities, and may be a subject in which most students feel disadvantaged. Therefore, there are more students who underestimate their mathematical efficacy than overestimate it.

By analyzing how the relationship between discrepancy in mathematics efficacy and mathematics anxiety changed according to bias in estimating mathematics efficacy, discrepancy in mathematics efficacy of the high-school students tended toward under- rather than over-estimation. When students underestimate their mathematics efficacy, their mathematics anxiety is also higher than in cases of overestimation. When students are not confident in their mathematical ability, they are more likely to experience fear and panic in situations that require mathematical reasoning and problem solving. Therefore, without considering the magnitude of the discrepancy of mathematical efficacy, we conclude that overestimating mathematical efficacy is more beneficial to an individual's mental health. Simultaneously, the significant difference between the degree of discrepancy in mathematical efficacy and mathematical anxiety according to the polarities of mathematical efficacy estimation bias also indicates the need to study the moderating effect of mathematical efficacy estimation bias on the relationship between discrepancy in mathematical efficacy and mathematics anxiety.

### 4.2. The effect of mathematical performance deviation on mathematics anxiety: the moderating effect of mathematical performance estimation bias

The correlation analysis revealed a significant negative correlation between mathematics efficacy and mathematics anxiety, which is consistent with previous research conclusions (37). There was also a significant positive correlation between the degree of discrepancy in mathematics efficacy and mathematics anxiety, which represents a novel finding. Studies have shown that the self-assessed and actual mathematics efficacy are positively correlated. Hence, greater self-assessed mathematics efficacy should be associated with lower mathematics anxiety (38). The greater the discrepancy in mathematical efficacy, the more inaccurate the self-assessment of mathematical efficacy, and thus higher the mathematics anxiety. Therefore, without considering other factors, the ability of mathematics efficacy to predict mathematics anxiety may be affected by the discrepancy in mathematics efficacy and the bias in estimated mathematics efficacy. Judgments of mathematical efficacy are subjective and volatile; many middle-school students are unclear regarding their level of ability and may not be able to accurately accurately assess their mathematics efficacy. As such, predicting the mathematics anxiety of such persons would also be inaccurate. People who are overly modest may judge their math efficacy to be low, but be calm when actually responding to a math test; thus, their math anxiety is also low. People who are overly self-confident, may judge their mathematical efficacy to be very high, but when they actually take a test, they may become seriously anxious due to their lack of actual ability; hence, their mathematics anxiety is very high. Both of these cases reduce the ability of mathematics efficacy to predict mathematics anxiety. This revelaed that both such cases are common. Thus, it is necessary to consider the influence of the degree of discrepancy in rated vs. actual ability and estimation bias in detail.

The moderating analysis showed that, in the first-grade students, bias in mathematics efficacy estimation played a moderating role in the relationship between discrepancy in mathematics efficacy and mathematics anxiety. That is, the influence of mathematics efficacy on mathematics anxiety varies according to variation in efficacy judgments. The data of the first-year high-school students showed that when the students underestimated their mathematics efficacy, increasing discrepancy in mathematics efficacy was associated with greater mathematics anxiety. When students overestimated their mathematics efficacy, the degree of discrepancy in mathematics efficacy was unrelated to mathematics anxiety. Nonetheless, when the participants overestimated their mathematics efficacy, mathematics anxiety in the first graders showed a decreasing trend with increasing discrepancy in math efficacy. The results of this study are different from those of previous studies (26, 29, 30–32). This highlights the need to consider the moderating effect of bias in the estimation of mathematical efficacy when considering the effect of the discrepancy between perceived and actual mathematical efficacy on mathematics anxiety. High-school students might not have a clear understanding of their mathematical ability. Therefore, high-school students, especially those who underestimate their own mathematical efficacy, should be helped to correctly understand their ability, to improve their confidence in their actual mathematical ability, to not be excessively pessimistic about their own ability; this would reduce mathematics anxiety and thus promote psychological health.

In the second grade of senior high-school, mathematical efficacy estimation bias did not moderate the path between the degree of discrepancy in mathematical efficacy and mathematics anxiety. For both overestimation and underestimation, the impact of discrepancy in mathematical efficacy on mathematics anxiety was positive, which is consistent with previous studies. However, from the interaction effect graph, students who overestimated their mathematics efficacy were lower in mathematics anxiety than students who underestimated their mathematics efficacy. Therefore, the suggestion that improving mathematics efficacy can reduce mathematics anxiety is further supported. However, for the second-year high-school group, measures should be taken to help students calibrate their mathematical efficacy, which is the most beneficial method for developing students' psychological health. If the individual overestimates their self-efficacy, as the degree of overestimation becomes larger, but the actual mathematical ability does not reach the corresponding level, when faced with mathematics learning or test questions, the anxiety of the individual will increase. This is a case of having excessively high expectations that are not reflected in actual ability. This leads to disappointment in oneself, which fosters negative emotions. However, if the individual underestimates their sense of efficacy and continues to distrusting their own true ability, this low sense of efficacy will cause the individual to gradually quit learning and facing challenging mathematics problem. This results in a decrease in their actual ability, fostering anxiety, and poorer mathematics test scores.

In summary, this study suggests that different educational measures should be taken for students of different grades. For younger high-school students, overestimating their own mathematics efficacy is beneficial to relieve mathematics anxiety, while underestimating mathematics efficacy will lead to more serious mathematics anxiety. Therefore, it is necessary to help students improve their mathematics efficacy. For older high-school students, overestimating or underestimating mathematics efficacy will lead to an increase in the level of mathematics anxiety. Thus, it is necessary to help these students estimate their mathematics efficacy as accurately as possible.

## 5. Conclusion

This study permits the following conclusions to be drawn. First, mathematics anxiety is significantly negatively correlated with mathematics efficacy, and significantly positively correlated with the degree of discrepancy in mathematics efficacy, whereas mathematics efficacy is significantly negatively correlated with the degree of discrepancy in mathematics efficacy. Second, the degree of discrepancy in mathematics efficacy and mathematics anxiety were significantly different according to the bias in the estimation of mathematics efficacy. Finally, the degree of discrepancy in the mathematics efficacy of high-school students was predictive of mathematics anxiety, and was moderated by the bias in estimated mathematics efficacy.

## Data Availability

The datasets presented in this study can be found in online repositories. The names of the repository/repositories and accession number(s) can be found in the article/Supplementary Material.
